# Cyclin D1 overexpression is associated with poor prognosis in oropharyngeal cancer

**DOI:** 10.1186/1916-0216-42-23

**Published:** 2013-03-19

**Authors:** Rui Jun Lin, Tarinee Lubpairee, Kelly Y Liu, Donald W Anderson, Scott Durham, Catherine F Poh

**Affiliations:** 1 Division of Otolaryngology-Head and Neck Surgery, University of British Columbia, Vancouver, BC, Canada; 2 Integrative Oncology, BC Cancer Agency & Research Centre, Vancouver, BC, Canada

**Keywords:** Oropharyngeal squamous cell carcinoma (OpSCC), Human papillomavirus (HPV), p16, Cyclin D1, Disease free survival, Prognosis

## Abstract

**Objectives:**

To determine the biological characteristics of oropharyngeal squamous cell carcinoma (OpSCC) and related outcome.

**Design:**

Retrospective study.

**Methods:**

Patients (N=60) with primary OpSCC from 2000 to 2005 were retrospectively identified from Pathology database and the outcome was confirmed through chart review. Among these, 41 biopsy samples with enough tissues were retrieved to construct a tissue microarray for detection of the presence of high-risk human papillomavirus (HPV) using Chromogenic *in situ* hybridization (CISH) as well as the expression of p16 and cyclin D1 using immunohistochemistry.

**Main outcome measures:**

Disease-free survival.

**Results:**

Among 60 patients, 39 (65%) patients had no recurrence or died without disease at the last follow-up (disease-free survival or Group 1), and 21 (35%) patients had persistent disease or died of disease (progression-free survival or Group 2). Although follow-up time was twice as long in group 1 (4.7 ± 2.2 vs. 2.0 ± 1.6 years; *P* < 0.0001), there was no difference between the 2 groups in age, gender, smoking/alcohol habits, TNM staging and treatment modalities. Among those 41 cases with available tumour tissues, there was no difference in HPV status and p16 expression between the 2 groups but a significant difference in cyclin D1 expression (*P* = 0.05). Using Kaplan-Meir survival analysis and log-rank test, cyclin D1 overexpression was highly associated with a poor prognosis when comparing time to outcome (*P* < 0.0001).

**Conclusion:**

Cyclin D1 overexpression is a potential prognostic marker of OpSCC.

## 

The incidence of oropharyngeal squamous cell carcinoma (OpSCC), particularly those arising in the base of the tongue and in the tonsillar region, rises every year between 1973 and 2004 [1]. This increasing trend occurs despite the decrease in the incidence of oral cavity, laryngeal and hypopharyngeal cancers as well as the decreased prevalence in smoking, which is a primary risk factor for these cancers [2]. Molecular studies have identified human papillomavirus (HPV) as a causative agent in 60% to 80% of patients with OpSCC [3]. HPV-16 accounts for a large majority of HPV-positive OpSCC (80-90%) compared to other oncogenic types such as HPV-18, 31 or 33 [4,5].

HPV-positive OpSCCs have distinct risk factors and molecular profiles. These tumours are thought to be associated with certain types of sexual behavior, but not to tobacco smoking or alcohol use. On a molecular level, almost all HPV-positive tumours express wild-type p53 tumour suppressor genes. Expression of HPV viral E6 and E7 oncoproteins inactivates p53 and the retinoblastoma protein pRb. p16 protein expression is subsequently elevated due to loss of the pRb negative feedback loop [6]. p16 then inhibits cyclinD1-CDK4/6 (cyclin-dependent kinase 4/6) complexes, which are important regulators for the cell cycle to progress from the G1 phase to the S phase. As a result, HPV-positive cancers are thought to be associated with downregulation of cyclin D1 expression [7,8]. In other head and neck cancers that are not typically related to HPV, cyclin D1 overexpression has been linked with poor outcome, particularly in hypopharyngeal cancers [9]. However there is not a large amount of evidence suggesting its prognostic significance in oropharyngeal cancer.

Given the worldwide growing epidemic of HPV-associated oropharyngeal cancers, characterization of HPV-associated molecular markers is becoming increasingly important for determining their impact on the prognosis of disease and for guiding the development of new therapeutic interventions. The objective of our study was to determine the association of cyclin D1 and HPV status in OpSCC and its relevance on patient outcome. Since cyclin D1 overexpression has been associated with a poor prognosis in cancers of other head and neck regions, we hypothesize it is associated with a poor prognosis in oropharyngeal cancer.

## Methods

### Patient and tumour tissue

This is a retrospective case series. The study has obtained approval from the Research Ethics Board at the University of British Columbia and has been in compliance with research conduct guidelines at this institution. A total of 60 patients (n = 60) with primary OpSCC from the year 2000 to 2005 were retrospectively identified from the Vancouver General Hospital Pathology database. This duration was chosen so that all patients have had at least 5 years of follow-up after their initial treatment. These patients were confirmed to have no previous history of cancer and their outcome information were collected through electronic chart review from the patient information database at the British Columbia Cancer Agency (BCCA). Demographic and clinical data were also collected from chart review, including age, gender, smoking status, alcohol intake, treatment received, follow-up time and survival status. Tumour staging using the TNM classification [10] was determined by review of clinical notes, radiology reports, surgical notes and pathology reports. Among the included patients, 41 (68%) formalin-fixed biopsy specimens with enough tissues were retrieved from the pathology archive for the construction of tissue microarrays.

### Tissue microarray (TMA) construction

Tissue microarray was constructed as previously described [11]. The most representative areas of tumours were chosen by the pathologist (CFP). Tissue cores of 0.6 mm in diameter from these representative areas of formalin-fixed pffin-embedded (FFPE) tumour blocks were taken by a manual tissue arrayer (Beecher Instrument, SI, USA). The cores were then transferred in duplicates to the recipient TMA block. The recipient blocks were then serially cut into 4 μm tissue sections for chromogenic *in situ* hybridization and immunohistochemistry.

### Chromogenic *in situ* Hybridization (CISH) for HPV high-risk subtypes and HPV-16/18

High-risk HPV subtypes and specifically HPV subtypes 16 as well as 18 in FFPE TMA sections were detected by using chromogenic *in situ* hybridization (CISH). Two HPV probes were examined in this study: HPV-HR (cocktail for high-risk types 16, 18, 31, 33, 35, 39, 45, 51, 52, 56, 58, 59, and 68, DAKO, Denmark), and HPV-16/18 (DAKO, Denmark). In brief, each of the tissue sections were depffinized, rehydrated and pretreated with target retrieval solution (DAKO) for 20–40 minutes at 97°C. After antigen retrieval with Proteinase K (Sigma-Aldrich, Canada) and blocking, the slides were hybridized with a biotinylated HPV-HR probe and HPV-16/18-specific probe. After initial binding of streptavidin horseradish peroxidase complex to the probe, signal amplification was performed using biotinyl tyramide. Positive hybridization signals were visualized by adding the chromogenic substrate diaminobenzidine (DAB). SiHa and SCC-9 cell lines were used as a positive and negative control, respectively. Slides were scored positive for HPV if a nuclear, punctuate pattern of signals was observed in > 60% of the tumour nuclei, which was indicative of integrated HPV DNA into the host genome. The scoring was performed by the same pathologist (CFP).

### Immunohistochemistry (IHC) for p16 and cyclin D1

IHC was performed on TMA tissue sections that were depffinized. After target retrieval, sections were incubated with primary antibodies for p16 (Roche mtm laboratories AG, Heidelberg, Germany) [12] and cyclin D1 (Cell Signaling, Ontario, Canada) [13] with duration and temperature specified for each marker (Table 1). The sections were then incubated with the EnVision+ System (DAKO Corporation) for cyclin D1 for detection of antigen-antibody reactions. Sections were visualized with DAB and counterstained with Mayer’s hematoxylin. The staining intensity was scored as a percentage of stained tumour cells (0% - 100%). Tumour cells with a greater than or equal to 50% staining percentage were scored as “positive”. Otherwise they were scored as “negative”. The scoring was again performed by the same pathologist (CFP).

**Table 1:** Antibodies used in immunohistochemistry for p16 and cyclin D1

**Marker**	**Dilution**	**Incubation**	**Staining pattern**	**Positive control**	**Negative control**	**Catalogue number**
p16	Ready to use	Room temperature, 30 min	Nuclear and cytoplasmic	SiHa	Muscle	CINtec® Histology Kit (Roche mtm laboratories AG, Heidelberg, Germany)
Cyclin D1	1:35	4°C, Overnight	Nuclear	SCC-9	Muscle	Cyclin D1 (92G2) Rabbit mAb #2978 (Cell Signaling, Ontario, CA)

### Statistical analysis

All statistical analyses were performed using SPSS/PASW Statistics (version 19, SPSS Inc., Chicago, IL). The Fisher’s exact test was used to compare dichotomous (e.g., gender, alcohol consumption and N staging) or other categorical (e.g., smoking status, T staging, TNM staging, and treatment) variables. Student’s *t*-test was used to compare pmetric variable (e.g., age and follow-up time). Disease-free survival was estimated using the Kaplan-Meier method with log-rank test for determining statistical significance. Disease-free survival was defined as time from the day of cancer diagnosis to the date of death from OpSCC or to the date of last follow-up. Multivariate analysis was not performed given the small sample size of the study. A *P* value of 0.05 or less was considered statistically significant.

## Results

### Patient population

Seventy-seven patients were initially identified to have primary oropharyngeal squamous cell carcinoma from the year 2000 to 2005 from the Pathology database of the Vancouver General Hospital. Seventeen patients were excluded from the study after reviewing the electronic charts at the BC Cancer Agency. Reasons of exclusion included (1) primary tumours not at oropharyngeal site (n = 10); (2) no definitive treatment received due to patient refusal or palliation (n = 6); and (3) loss of follow-up (n = 1). A total number of 60 patients were included for the final analysis. The patient characteristics are shown in Table 2. The mean follow-up time was 4 ± 2.4 years. The patients were categorized into two outcome groups based on disease (OpSCC) status after treatment: Group 1 represented disease-free survival; Group 2 represented progression-free survival or disease-specific deaths. Among the 60 patients, 39 (65%) had no recurrence or died without disease (Group 1) and 21 (35%) had persistent disease or died of disease (Group 2). There was no significant difference among patient characteristics including age, smoking, alcohol intake, and TNM staging between the two outcome groups (Table 2). Follow-up time was significantly longer for Group 1 compared to Group 2 (4.7 ± 2.2 years vs. 2.0 ± 1.6 years; *P* < 0.0001).

**Table 2:** Comparison of patient characteristics between two outcome groups

	**Total (N = 60)**	**Group 1**^**a **^**(N=39, 65%)**	**Group 2**^**a **^**(N = 21, 35%)**	***P *value**
**Mean age at diagnosis (yr ± SD)**	60.4 ± 11.7	59.8 ± 11.3	61.4 ± 12.6	0.63
**Gender**				
Male	46 (77)	30 (77)	16 (76)	
Female	14 (23)	9 (23)	5 (24)	1
**Smoking Habit**				
Non-smokers	14 (23)	10 (26)	4 (19)	0.78
Former smokers (quit > 5 yr)	15 (25)	9 (23)	6 (29)	
Current smokers	29 (48)	20 (51)	9 (43)	
Unknown^e^	2 (3)	0 (0)	2 (10)	
**Alcohol**^**b**^				
Never or light	51 (85)	34 (87)	17 (81)	1
Heavy	7 (12)	5 (13)	2 (10)	
Unknown^e^	2 (3)	0 (0)	2 (10)	
**T staging**				
1	25 (42)	19 (49)	6 (29)	0.44
2	15 (25)	9 (23)	6 (29)	
3	12 (20)	6 (15)	6 (29)	
4	3 (5)	2 (5)	1 (5)	
Unknown^e^	5 (8)	3 (8)	2 (10)	
**N staging**				
N0	7 (12)	6 (15)	1 (5)	0.4
N+	51 (85)	32 (82)	19 (90)	
Unknown^e^	2 (3)	1 (3)	1 (5)	
**TNM staging**^**c**^				
1	2 (3)	2 (5)	0 (0)	0.61
2	4 (7)	3 (8)	1 (5)	
3	11 (18)	6 (15)	5 (24)	
4	41 (68)	27 (69)	14 (67)	
Unknown^e^	2 (3)	1 (3)	1 (5)	
**Treatment information**				
Surgery (S/T) only	3 (5)	2 (5)	1 (5)	0.12
Radiation (XRT) only^d^	30 (50)	18 (46)	12 (57)	
Chemotherapy only	11 (18)	5 (13)	6 (29)	
Combined S/T and XRT	16 (27)	14 (36)	2 (10)	
**Mean Follow-Up Time (yr ± SD)**	4 ± 2.4	4.7 ± 2.2	2 ± 1.6	< 0.0001

^a^Outcome: Group 1, those survived with no disease or died due to other cause (disease-free survival); Group 2, those alive with disease or died of disease (progress-free survival or disease-specific deaths).

^b^Heavy alcohol intake was defined as > 3 drinks/day for males and > 2 drinks/day for females.

^c^TNM staging is based on the American Joint Committee on Cancer (AJCC) Cancer Staging Manual. 7th ed 2010.

^d^Radiation only include radiation only with/without induction chemotherapy.

^e^Missing values (Group 1 (N): Group 2 (N)): Smoking Habit (0:2), Alcohol (0:2), T-staging (3:2); Nodal Status (1:1); TNM staging (1:1).

### HPV status and p16 expression

**Figure 1:**
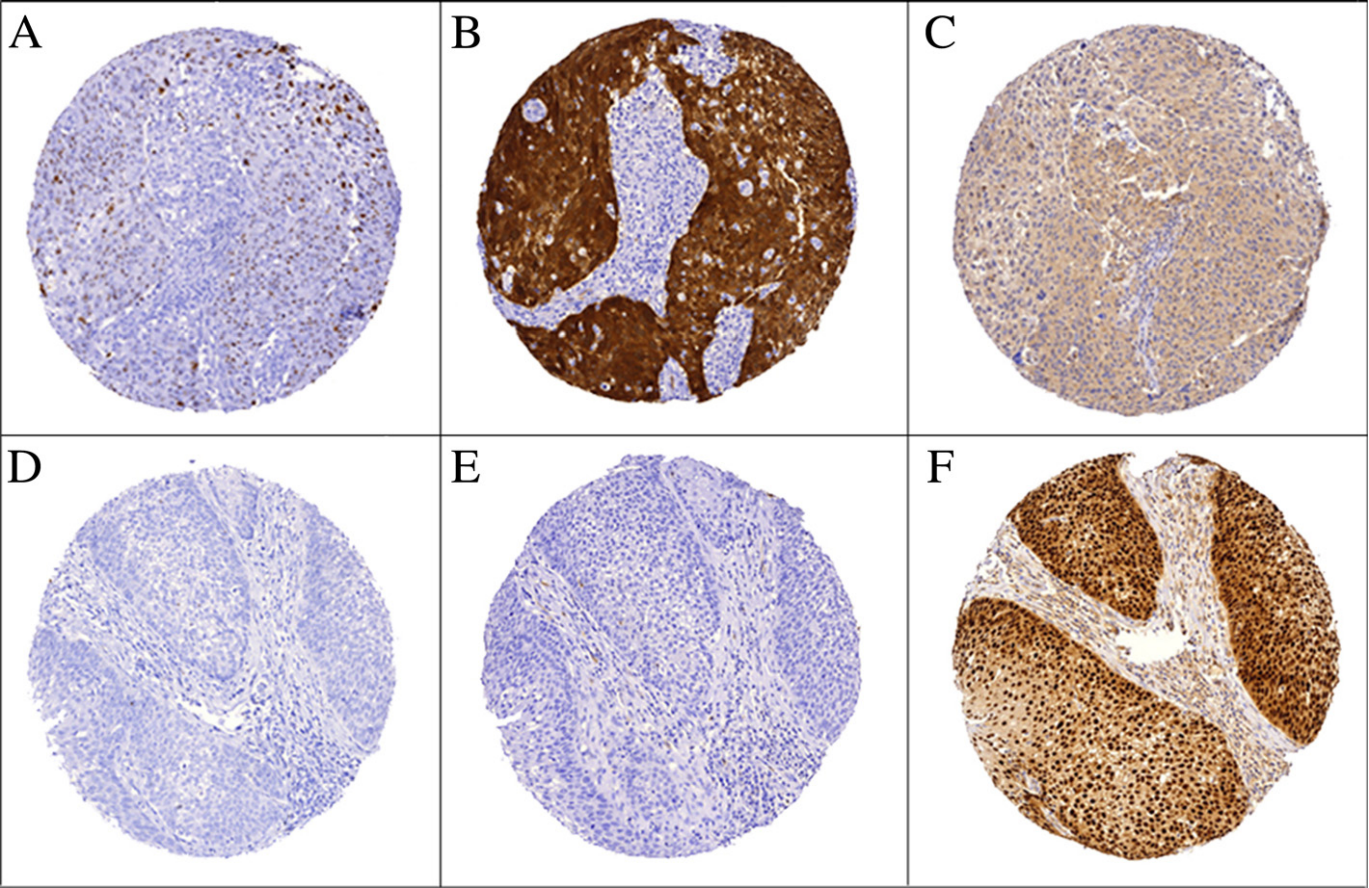
**Examples of a case from Group 1 (favourable outcome group; upper panel, A, B, C) and a case from Group 2 (adverse outcome group; lower panel, D, E, F). A** (diffuse punctate signals) and **D** (negative) showing CISH staining of HPV-HR DNA; **B** (diffuse strong cytoplasmic staining and nuclear staining) and **E** (negative) showing IHC staining of p16 protein; **C** (negative) and **F** (diffuse strong nuclear staining) showing IHC staining of cyclin D1. (Original magnification 100×).

Of the 60 patients included in the study, 41 had sufficient tumour tissues for analysis. Among these, 56% (n= 23) tested positive for high risk HPV DNA (HPV-HR) and 66% (n = 27) showed p16 overexpression. All cases that were HPV-HR-positive were also p16-positive (see examples in Figure 1). None of the tumours were HPV-positive and p16-negative. Approximately 9.8% of tumours were found to be HPV-negative and p16-positive. The sensitivity, specificity, positive predictive value and negative predictive value using p16 for the presence of HPV-HR DNA defined by CISH approach were 0.85, 1.0, 1.0, and 0.78, respectively. Among those cases that were positive for HPV-HR (n = 23), 68% were positive for HPV-16/18 DNA. While 7 out of 7 tumours from non-smokers were positive for p16, interestingly 20 out of 32 tumours from smokers (62.5%) were also positive for p16 (*P* = 0.08).

**Figure 2:**
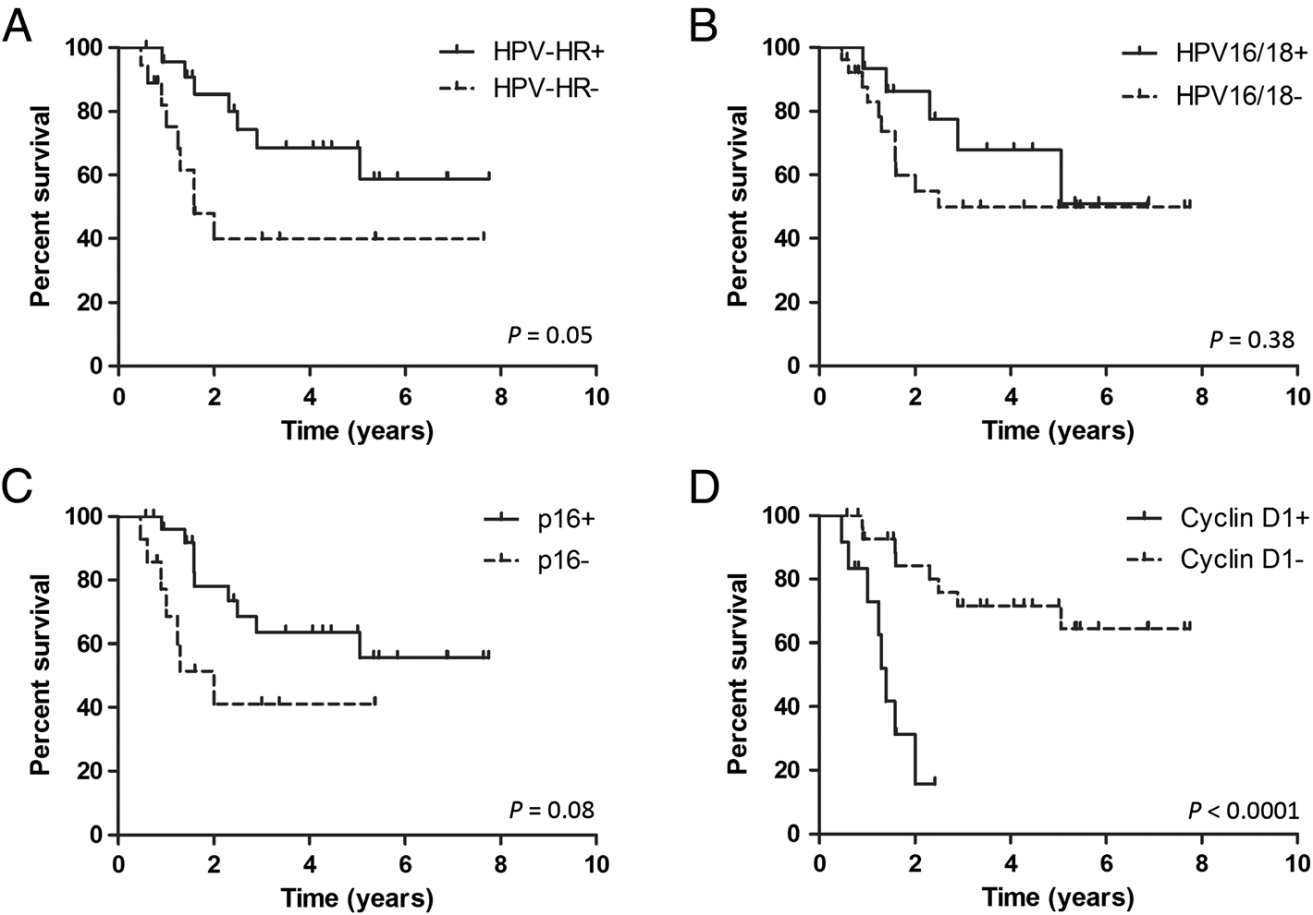
**Kaplan Meier analysis for disease-free survival and log-rank test for comparison between groups: A, HPV-HR (*P *=0.05); B, HPV-16/18 (*P *= 0.38); C, p16 (*P *= 0.08); and D, cyclin D1 (*P *< 0.0001).**

There was no difference in HPV status and p16 expression between Groups 1 and 2 (Table 3). However, disease-free survival was improved in tumours that were positive for HPV-HR (*P* =0.05, Figure 2A) compared to tumours that were positive for HPV-16/18 (*P* =0.38, Figures 2B). Although it was not statistically significant, the p16-positive group showed better disease-free survival than the p16-negative group (*P* = 0.08; Figure 2C).

**Table 3:** Comparison of tumour HPV DNA, p16 and cyclin D1 status between outcome groups 1 and 2

	**Total (N=60)**	**Group 1 (N=39, 65%)**	**Group 2 (N=21, 35%)**	***P *value**
**HPV-HR**				
Positive	23 (56)	14 (61)	9 (50)	0.54
Negative	18 (44)	9 (39)	9 (50)	
**HPV-16/18**				
Positive	15 (37)	8 (35)	7 (39)	1
Negative	26 (63)	15 (65)	11 (61)	
**p16**				
Positive	27 (66)	16 (70)	11 (61)	0.74
Negative	14 (34)	7 (30)	7 (39)	
**Cyclin D1**				
Positive	12 (29)	4 (17)	8 (44)	
Negative	29 (71)	19 (83)	10 (56)	0.09

### Cyclin D1 expression and disease-free survival

Twelve tumours were cyclin D1-positive (12/41, 29%) and twenty-nine tumours were cyclin D1-negative (29/41, 71%). Among the cyclin D1-negative tumours, 21 (78%) were positive for p16, a currently accepted surrogate marker for the presence of HPV DNA. For those tumours that were cyclin D1-positive (n = 14), only 43% (n = 6) were positive for p16. Using Fisher’s exact test, there was no difference in cyclin D1 expression between the two outcome groups (Table 3). However, the improved disease-free survival over time was significant in the cyclin D1-negative group using the log-rank analysis (*P* < 0.0001, hazard ratio 18.0, 95% confidence interval 4.37 to 74.03; Figure 2D).

## Discussion

Cyclin D1 overexpression has been reported in squamous cell carcinoma in a variety of head and neck subsites including the larynx, hypopharynx and tongue [9,14,15]. Its prognostic significance in OpSCC has been contradictory [16-22]. Our study has demonstrated cyclin D1 overexpression is associated with a poor prognosis in primary OpSCC. Cyclin D1 is a well-known oncogene in a variety of human cancers. It is a key regulator of cell proliferation. It promotes cell cycle progression through G1 phase by forming complexes with CDK4 and CDK6, which in turn phosphorylate the retinoblastoma protein pRb [23]. This causes pRb to release the E2F transcription factor, which then activates genes essential for cell cycle progression from G1 phase to S phase, during which cells proliferate [24]. It has been suggested that cyclin D1 plays a role in the late phase of tumour progression given its correlation with lymph node metastases, histological aggressiveness and poor prognosis [9].

Cyclin D1 overexpression was higher in patients with adverse outcomes in our study, but the difference was not statistically significant. With the log-rank analysis, however, the difference in disease-free survival over time was statistically significant between the cyclin D1-positive and cyclin D1-negative groups. Although this study had a small sample size, we fitted a multivariate Cox Proportional Hazard model to examine the potential joint and interaction effects among markers (data not shown). The analysis showed that cyclin D1 overexpression was the only and independent variable demonstrating significance in predicting poor outcome in disease-free survival. Despite the small number of patients in our study, our finding strongly suggest that cyclin D1 could potentially be a useful prognostic marker for OpSCC.

In our study, HPV DNA was detected in 56% of OpSCC with the entire 56% being p16-positive. This is consistent with the reported numbers from previous studies [25,26]. 9.8% of tumours were p16-positive and HPV-negative, which has also been reported in the literature [26]. Overall, our study supports p16 as an effective surrogate marker for high-risk HPV infection with high sensitivity and specificity (85% and 100%, respectively). Cyclin D1 expression should be downregulated in HPV-positive head and neck squamous cell carcinoma as a result of pRb suppression [7,24]. In our study, 78% (N=21) of cyclin D1-negative tumours were associated with p16 overexpression. This inverse relationship is consistent with the hypothesis that HPV positivity and subsequent p16 upregulation leads to suppression of cyclin D1 expression.

Our study has not found any significant association between smoking or alcohol intake and tumour HPV status. Interestingly, all the nonsmokers showed p16/HPV positivity but 62.5% of smokers have also shown p16/HPV positivity. This may argue against the current literature observation that HPV-positive tumours tend to occur in non-smokers and non-drinkers [27].

One of the limitations of this study is that it was a retrospective review, thus it relied on the accuracy of written or dictated medical records. A second limitation is the small sample size. We were only able to identify a total number of 60 cases of primary oropharyngeal carcinoma with known outcome from 2000 to 2005. Thus we may not have enough patients in order to detect a statistical significance in the expression of markers between the two outcome groups. We were also not able to perform a multivariate analysis due to the small sample size. The next step of this project is to widen our search through the BC Cancer Registry in order to identify more primary oropharyngeal cancer cases and to retrieve their tumour tissues for tissue microarray construction. A prospective arm of the study is also underway to recruit patients with new oropharyngeal carcinoma diagnosis to participate in our investigation.

## Conclusion

Our preliminary results have demonstrated cyclin D1 overexpression is a potential prognostic marker of primary oropharyngeal squamous cell carcinoma. Further understanding of these genetic alterations leading to malignant progression is critical to future secondary prevention strategies and therapeutic interventions for patients with HPV-positive oropharyngeal squamous cell carcinoma.

## Consent

Written informed consent was obtained from patients who participated in this study for publication of this report and any accompanying images.

## Competing interests

The authors declare that they have no competing interests.

## Authors’ contributions

RJL, SD and CFP designed the study protocol. RJL was responsible for ethics application, patient chart review, data collection and manuscript write-up. TL and KYL performed tissue microarrays, data collection and statistical analysis. DWA and SD were responsible for recruiting patients. CFP was the principle investigator overlooking the entire study. All authors read and approved the final manuscript.
